# Subcoronary versus supracoronary aortic stenosis. an experimental evaluation

**DOI:** 10.1186/1749-8090-6-100

**Published:** 2011-08-22

**Authors:** Mette Sorensen, J Michael Hasenkam, Henrik Jensen, Erik Sloth

**Affiliations:** 1 Department of Anaesthesiology and Intensive Care, Aarhus University Hospital, Skejby, Brendstrupgaardsvej 100, 8200 Aarhus N, Denmark; 2 Department of Cardiothoracic & Vascular Surgery, Aarhus University Hospital, Skejby, Brendstrupgaardsvej 100, 8200 Aarhus N, Denmark; 3 Institute of Clinical Medicine, Aarhus University Hospital, Skejby, Brendstrupgaardsvej 100, 8200 Aarhus N, Denmark

**Keywords:** subcoronary, supracoronary, porcine, aortic stenosis, myocardial hypertrophy

## Abstract

**Background:**

Valvular aortic stenosis is the most common cause of left ventricular hypertrophy due to gradually increasing pressure work. As the stenosis develop the left ventricular hypertrophy may lead to congestive heart failure, increased risk of perioperative complications and also increased risk of sudden death. A functional porcine model imitating the pathophysiological nature of valvular aortic stenosis is very much sought after in order to study the geometrical and pathophysiological changes of the left ventricle, timing of surgery and also pharmacological therapy in this patient group.

Earlier we developed a porcine model for aortic stenosis based on supracoronary aortic banding, this model may not completely imitate the pathophysiological changes that occurs when valvular aortic stenosis is present including the coronary blood flow. It would therefore be desirable to optimize this model according to the localization of the stenosis.

**Methods:**

In 20 kg pigs subcoronary (n = 8), supracoronary aortic banding (n = 8) or sham operation (n = 4) was preformed via a left lateral thoracotomy. The primary endpoint was left ventricular wall thickness; secondary endpoints were heart/body weight ratio and the systolic/diastolic blood flow ratio in the left anterior descending coronary. Statistical evaluation by oneway anova and unpaired t-test.

**Results:**

Sub- and supracoronary banding induce an equal degree of left ventricular hypertrophy compared with the control group. The coronary blood flow ratio was slightly but not significantly higher in the supracoronary group (ratio = 0.45) compared with the two other groups (subcoronary ratio = 0.36, control ratio = 0.34).

**Conclusions:**

A human pathophysiologically compatible porcine model for valvular aortic stenosis was developed by performing subcoronary aortic banding. Sub- and supracoronary aortic banding induce an equal degree of left ventricular hypertrophy. This model may be valid for experimental investigations of aortic valve stenosis but studies of left ventricular hypertrophy can be studied equally well by graduated constriction of the ascending aorta.

## Background

Valvular aortic stenosis is the most common heart valve disease and the development is often initiated by fibrosis and the subsequent development of calcification of the aortic leaflets and annulus [1].

Severe valvular aortic stenosis eventually leads to left ventricular failure characterized by diminished stroke volume due to increased pressure work and compensatory increase in end diastolic volume and pressure. The increased pressure work causes left ventricular hypertrophy and increased left ventricular mass. Although this hypertrophy enables some compensation for the pressure induced increase in wall stress according to the law of Laplace [2], it is associated with increased morbidity and mortality [3,4] together with increased risk of perioperative complications [5] when the aortic valve is replaced.

To evaluate the impact of different treatment options and developing new ones a human compatible animal model of valvular aortic stenosis is necessary.

Earlier attempts have been made to develop animal models for left ventricular hypertrophy [6-15]. Most of them are models for supracoronary or supravalvular aortic stenosis mainly rodent, porcine, canine and sheep models. To our knowledge there are no existing experimental porcine models for aortic stenosis based on subcoronary aortic banding. In our institution Lunde et al. [16] developed a porcine model for left ventricular hypertrophy by banding the ascending aorta downstream of the coronary ostia. This method creates a model that imitates the condition of aortic coractation or arterial hypertension rather than specifically valvular aortic stenosis.

Since, the pressure is elevated proximally to the stenosis the coronary vessels will be exposed to elevated systolic blood pressure and may cause increased systolic myocardial perfusion and potentially hampered diastolic perfusion of the myocardium. It may also enhance the development of left ventricular hypertrophy.

Due to these model limitations in the supracoronary stenosis setting it is desirable to develop an animal model that more closely imitates the pathophysiological scenario of valvular aortic stenosis characterized by slow progression of the stenosis in an animal that shares essential anatomical features with the human heart.

The aim of this study was threrfore to develop and evaluate a human compatible porcine model for valvular aortic stenosis and compare it to supracoronary aortic stenosis.

## Methods

### Study design

The study was conducted as a prospective randomized intervention-control study with three groups of female Danish Landrace pigs.

In the subcoronary group (n = 8) aortic banding was performed at the level of the aortic valve. In the supracoronary group (n = 8) aortic banding was made in the mid ascending aorta and in the control group (n = 4) a sham operation was performed in order to obtain normal values for pigs at the same stage of development with almost the same surgical trauma.

Prior to surgery the pigs were randomized to one of the three study arms. Preoperative transthoracic echocardiography was conducted and the surgical banding procedure or sham operation performed subsequently. At four, six and eight weeks postoperatively the echocardiography was repeated and at the last follow-up examination at eight weeks the blood flow in the left anterior descending coronary artery was also measured intraoperatively prior to euthanasia of the animal.

### Animal preparation and anesthesia

In order to include the scheduled number of animals 44 Danish Landrace pigs weighing approximately 20 kg (19.89 ± 1.7 kg) were included in this study.

Each animal was pre-anaesthized prior to transport from the housing facility with an intra muscular injection of 40 mg azaperone (Stresnil^®^) and 10 mg midazolam (Dormicum^®^).

Immediately upon arrival to the surgical facility i.v. access was obtained in an ear vein and 20 mg midazolam (Dormicum^®^) and 40 mg ketamin were administered intravenously.

The animal was then endotracheally intubated and then coupled to a ventilator. Before commencing surgery 4 mg pancuronium and 750 mg cefuroxim (Zinacef^®^) were administered intravenously. During the banding procedure continuous anesthesia and analgesia were maintained by isoflurane/sevoflurane aiming at a constant MAC = 1.0 and 350 μg/hour fentanyl (Haldid^®^). After the operation cefuroxim (Zinacef^®^) 750 mg were administered intravenously and 100 mg of the NSAID Flunixin vet. ^® ^intra muscularly.

At the follow-up examinations the animals were pre-anesthetized as at the primary operation and the echocardiography was performed. At the last follow-up the anesthesia was identical to the one performed at baseline.

### Surgical procedure

In all groups a left lateral mini-thoracotomy was made under sterile conditions in the third intercostal space to display the lung which was then carefully pushed aside to expose the most cranial part of the heart.

In the subcoronary group the blood pressure was recorded in the descending aorta in order to monitor the systemic blood pressure during the operation. Then the left coronary artery main stem was carefully mobilized as was the most proximal parts of the left anterior descending artery and the circumflex artery. The right coronary artery was then mobilized and the most proximal part carefully dissected. A thin silicone tube was threaded with a Ticron 2-0 suture and placed around the aorta, upstream of both coronary arteries and the suture was tied in a tight but non-restrictive fashion.

In the supracoronary group a threaded silicone tube was placed around the ascending aorta. It was then approximated to the aorta in a non-constricting fashion as in the subcoronary group.

To ensure compatibility between the two intervention groups and the control group a sham operation was performed in the latter group. Instead of banding the aorta a silicone tube with the length of approximately 15 mm was sutured to the connective tissue between the ascending aorta and the pulmonary artery. In this manner the animals in the control group achieved the same foreign body reaction and their growth rate also suffered the same decline due to the surgical trauma.

In all groups the pericardial sack was left open and the ribs were approximated with a 0 Polysorb^® ^suture. Finally, the skin was closed using Biosyn^® ^3-0 and the pneumothorax was evacuated to avoid the need of pleural drainage.

The animal was ear tagged, extubated and transported back to the housing facility when awake and hemodynamically stable.

### Follow-up examinations

At each follow-up examination the animal was anesthetized, weighed and an echocardiography was performed.

The examinations were performed using a 3.5 MHz matrix transducer coupled to a VIVID 7 ultrasound machine (GE Healthcare, Horten, Norway).

Left ventricular free wall thickness was assessed through a right transthoracic acoustic window using the parasternal long axis scan. From the echocardiographic loops the left ventricular wall thickness was assessed using dedicated software (Echopac GE Healthcare, Horten, Norway). An anatomical M-mode assessment was applied to the loops of the parasternal long axis scan and the free wall thickness measured with the examiner being blinded for which animal was examined and also with respect to the week which the examination was performed.

The coronary artery blood flow was measured using a transit time CardioMed flow probe mounted on the left anterior descending artery at the level of the second diagonal. The data was collected using LabView 8.2 (National Instruments, Texas, USA) and also analyzed using this software. After mounting the flow probe the measurements was obtained after approximately 30 minutes of rest when the heart rate and blood pressure were stable.

According to the blood pressure measurements the heart cycle was divided into the systolic and the diastolic part marked by the closing of the aortic valve. The coronary blood flow signal was then integrated and the ratio of the systolic and the diastolic blood flow, blood flow ratio was derived, BRF = Q_syst_/Q_diast_.

The entire heart was excised and then fixated in formaldehyde for several weeks before further examinations. At this point the weight of the left ventricle was assessed and the apical 6 cm of the heart was cut in slices and the right ventricle removed. The heart/body weight ratio was calculated as H/BWR = (Heart weight (kg)/Body weight (kg)) × 10^3^.

The primary endpoint was left ventricular free wall thickness and the secondary endpoints were post-mortem heart/body weight ratio and blood flow ratio in the left anterior descending.

### Ethical considerations

Qualified and experienced animal caretaker personnel monitored the health status of the animals during the study period. The experiments complied with the guidelines for animal experimental studies issued by The Danish Inspectorate for Animal Experimentation under the Danish Ministry of Justice who also approved this study and the investigation conforms to the *Guide for the Care and Use of Laboratory Animals *published by the US National Institutes of Health [NIH Publication No. 85-23, revised 1996].

During the development of aortic stenosis the animals were carefully evaluated for signs of heart failure and in case of failure-to-thrive they were electively euthanized.

By the end of the final follow-up at eight weeks the animals were euthanized by an intra cardiac injection of saturated potassium solution during general anesthesia.

### Statistical evaluation

The data from the three groups was compared using one way ANOVA-test. The data was normal distributed verified by Q-q plot and a scatter plot.

If a p-value < 0.05 was found using the one way ANOVA, post hoc testing was performed using an unpaired t-test comparing the groups two-by-two.

p-values < 0.05 were considered statistically significant. Results are displayed as mean ± standard deviation.

## Results

### Survival

Out of the 44 animals included in this study 10 pigs were excluded because of pre-existing disease mainly pneumonia and/or pericarditis (9) and malformation of the larynx (1). During surgery 14 died including 12 from the subcoronary group (10 due to ischemia and/or arrhythmias, 2 because of uncontrollable bleeding) and 2 from the supracoronary group because of postoperative ventricular fibrillation. All surviving animals (8 pigs in the subcoronary group, 8 pigs in the supracoronary group and 4 pigs in the control group) completed the follow-up period. Full data sets were acquired in 18 animals. In two animals, one from each of the supracoronary and control group, coronary blood flow measurements were not completed due to uncontrollable bleeding when mobilizing the heart and mounting the flow probe.

**Figure 1:**
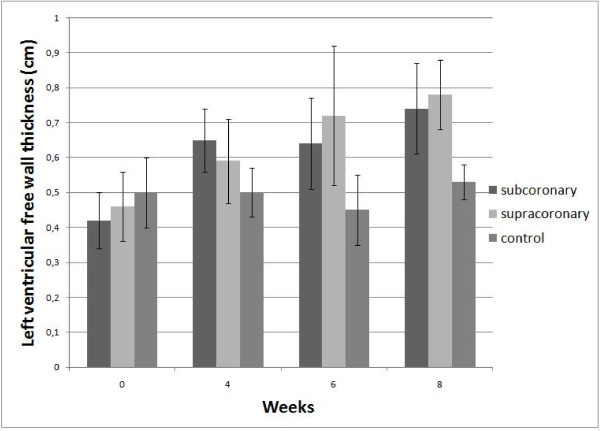
**Left ventricular free wall thickness**.

The results are displayed in Figure 1 and tables 1 and 2 and show that both subcoronary and supracoronary aortic banding can induce left ventricular hypertrophy compared with controls measured by echocardiography and also by post mortem examinations of the left ventricle.

**Table 1:** Heart/body weight ratio

Subcoronary	Supracoronary	Control	**p**_**ANOVA**_
2.34 ± 0.28	2.58 ± 0.42	1.79 ± 0.24	0.0055

**Post hoc testing**			**p-values**
Subcoronary group vs. supracoronary group:			0.20
Subcoronary group vs. control group:			0.008
Supracoronary group vs. control group:			0.006

**Upper part: **Heart/bodyweight ratio. **Lower part: **p-values from post hoc testing using unpaired t-test.

**Table 2:** Coronary blood flow ratio

**BFR = Blood Flow**_**systolic **_**/Blood Flow**_**diastolic**_				
	**Subcoronary**	**Supracoronary**	**Control**	**P**_ANOVA_

**Average**	0.36 ± 0.10	0.45 ± 0.25	0.34 ± 0.22	0.36

**Heart rate 50-55 BMP**	0.27 ± 0.095	0.55 ± 0.35	0.39 ± 0.18	0.42

**Blood Flow Ratio. Upper part: **Average for the groups. **Lower part: **Flow data for the heart rate interval 50-55 BPM.

### Left ventricular free wall thickness

The increase in wall thickness was evident after only four weeks in the two intervention groups compared with the control group but the increase was not statistically significant until after six weeks and stayed clearly hypertrophic through the eight weeks examination when comparing them to the control group. There were no statically significant differences between the two intervention groups.

### Coronary blood flow ratio

The coronary blood flow measurements revealed a slightly higher systolic blood flow ratio in the left anterior descending artery in the supracoronary group than the subcoronary and the control group but not statistically significant different when testing with a one way ANOVA test. The data was stratified according to the heart rate and the only interval in which all groups were represented was 50-55 BPM. We also pooled the data and calculated a flow average for all three groups including all data on surviving animals in which all flow measurements are represented meaning that the data is not stratified according to heart rate. See table 2.

## Discussion

This study shows that it is possible to perform subcoronary aortic banding and induce left ventricular hypertrophy based on human compatible valvular aortic stenosis. The results from the echocardiographic examinations indicate that the left ventricular hypertrophy parallels the gradually developing stenosis.

The increase in wall thickness in the subcoronary group proceeds in an almost linear fashion with some degree of stagnation at six weeks. The increase in wall thickness in the supracoronary group proceeds in a linear fashion. In the control group there is almost no increase in wall thickness though showing a similar pattern with a slight decrease in wall thickness at six weeks as in the subcoronary group. This growth pattern might be within the normophysiological growth of the porcine heart since it also occurred in the control group and has been described in other studies [16]. Since we chose the development of left ventricular hypertrophy as our primary endpoint we did not measure the pressure drop across the stenosis since the resistance was obviously hemodynamically significant.

The post mortem examinations revealed significant variation in the weight of the left ventricle as well as the total weight of the animals. To circumvent this problem we related the heart weight to the body weight (heart/body weight ratio) and were able to perform a less biased comparison.

We found that the heart/body weight ratio was significantly increased in the intervention groups compared with the control group. No significant differences were found between the two intervention groups. This was supported by the echocardiographic findings.

The blood flow ratios showed a tendency towards a higher systolic flow in the left anterior descending artery in the supracoronary group compared with the subcoronary and the control group. There was, however, no statistical difference when testing both the average flow ratio and the ratio for the flow ratio interval 50-55 BPM which was the only narrow heart rate interval where all groups was represented. The low number of animals included in this study may explain the lack of significance. The results may however, still reflect a tendency towards different hemodynamic conditions according to the location of the stenosis meaning that there could be increased systolic myocardial perfusion in the supracoronary group compared with the subcoronary group. On the other hand, it did not seem to affect the development of left ventricular hypertrophy in the two intervention groups.

The results indicate that it is possible to induce left ventricular hypertrophy using subcoronary banding technique. It also suggests that the blood flow in the left anterior descending artery is only slightly influenced by the localization of the aortic stenosis since the blood flow ratio only is slightly higher in the supracoronary group.

Animal models producing pure valvular aortic stenosis are very scarce and only a canine model has been presented in the literature [13]. To our knowledge no study has been performed using subcoronary aortic banding in a porcine model and with this study we have shown that it is technically feasible to create a human compatible porcine model for valvular aortic stenosis.

The mortality levels in the subcoronary group were high. The reason for this may be that there was a learning curve for the subcoronary group since the surgery needed to perform the procedure was more complex than the surgery performed in the two other groups. The procedure in this study was performed via a left lateral mini-thoracotomy. This made the subcoronary aortic banding technically challenging. The mortality levels could possibly have been improved if the aortic banding had been performed via a median sternotomy. Using this access would inflict the animals with a more extensive surgical trauma and an increased risk of postoperative complications.

Since supracoronary aortic banding is easier accessible and entails a lower mortality and almost certainly will result in left ventricular hypertrophy it may be more practical and less demanding to use this method.

In future studies investigating left ventricular hypertrophy the supracoronary aortic banding method will be appropriate since subcoronary- and supracoronary aortic banding creates the same degree of hypertrophy and this method is easier accessible. In studies investigating the nature of valvular aortic stenosis the animal model should closely imitate the nature of that and therefore the subcoronary method should be used. Therefore, it may be relevant to consider the type of banding when investigating left ventricular hypertrophy or valvular aortic stenosis.

Since congenital subcoronary aortic stenosis initially may be asymptomatic and may lead to the development of left ventricular hypertrophy and congestive heart failure it is important to establish an animal model that closely imitates this condition. The model for subcoronary aortic stenosis may be appropriate for studying congenital aortic stenosis and evaluating different treatments such as timing of surgery, choice of procedure and long-term outcome after treatment or without treatment.

### Study limitations

The difference between the two intervention groups and the control group might have been accentuated by doing this study in a larger scale with respect to the number of included or surviving animals.

The statistics regarding the blood flow measurements are not very strong and this study must be regarded as a feasibility study in which we proved that it is possible to perform subcoronary aortic banding.

The flow ratio data was difficult to compare due to different heart rates while collecting the flow measurements. This may have caused some degree of bias because a higher heart rate causes the diastolic part of the heart cycle to decrease thereby causing a higher ratio.

It could have been desirable but impractical to measure the myocardial wall thickness by the transthoracic echocardiographic measurements at several locations in the left ventricle. Therefore, we can only anticipate that the growth is concentric. However, other studies have shown that left ventricular hypertrophy caused by valvular aortic stenosis is concentric [2] and it is reasonable to assume that this is the case here also. We included animals with a body weight of 20 kg. The reason for not using smaller animals that would develop a higher degree of stenosis at the banding point -since the aorta is fixed at a smaller diameter- is the complex surgery needed for performing subcoronary aortic banding. The banding becomes a little less challenging when using animals weighing 20 kg than it would have been in smaller animals.

Another option that may improve this model is extending the follow-up period allowing the animals to grow even larger and also allowing the left ventricular hypertrophy to develop to a greater extent. However, an extended follow-up period may also challenge the model since congestive heart failure may arise.

## Conclusion

A human pathophysiological compatible porcine model for valvular aortic stenosis was developed by performing subcoronary aortic banding. Comparing the combined subcoronary and the supracoronary groups with the control group we found significantly increased left ventricular free wall thickness, significantly increased heart/body weight ratio and no statistically significant difference in the coronary blood flow ratio. There were no statistical differences between the sub- and supracoronary group when testing each endpoint.

Unless future studies should disclose any qualitative differences between left ventricular hypertrophy induced by subcoronary and supracoronary aortic banding it may be most practical to use the supracoronary banding approach when studying left ventricular hypertrophy.

### Perspectives

This chronic porcine model of valvular aortic stenosis may very well form the basis for a wide range of experimental settings. With this human compatible pathophysiological animal model it is possible to investigate the clinical challenges entailed by this patient group and the nature of left ventricular hypertrophy due to valvular aortic stenosis. This model is suitable for evaluating new bioprosthesis and the potential impact that prosthesis-patient mismatch may have on the valve such as calcification and rupture of the valve but also the consequences of diminished regression of the left ventricular hypertrophy can be studied using this model. It also becomes possible to evaluate novel pharmacological therapies aimed at relieving valvular aortic stenosis and left ventricular hypertrophy in adult as well as pediatric patients. In addition, the model can be used to investigate how the timing of valve surgery can be optimized in this patient group.

## Competing interests

The authors declare that they have no competing interests.

## Authors' contributions

MS participated in the design of the study and carried out the experimental work, data analysis and drafted the manuscript. JHM participated in designing the study, coordinating the experimental surgeries and the statistical evaluation. HJ participated in the experimental surgeries. ES participated in designing the study and collected the echocardiographic follow-up examinations. All authors read and approved the manuscript.
